# Vertical jump neuromuscular performance of professional female handball players—starters vs. non-starters comparison

**DOI:** 10.3389/fspor.2024.1407601

**Published:** 2024-05-09

**Authors:** Katarina Radovic, Dimitrije Cabarkapa, Jelena Aleksic, Damjana V. Cabarkapa, Dragan M. Mirkov, Olivera M. Knezevic, Andrew C. Fry

**Affiliations:** ^1^ Faculty of Sport and Physical Education, University of Belgrade, Belgrade, Serbia; ^2^ Jayhawk Athletic Performance Laboratory—Wu Tsai Human Performance Alliance, Department of Health, Sport and Exercise Sciences, University of Kansas, Lawrence, KS, United States

**Keywords:** force, power, velocity, handball, testing, athlete, biomechanics

## Abstract

Given the complex nature of the handball as a game, players are required to possess a distinct set of physical and physiological attributes to attain peak performance. With the countermovement vertical jump (CVJ) being widely implemented as a non-invasive and time-efficient testing modality in sports settings, the purpose of the present study was twofold: (a) to establish a CVJ profile of professional female handball players and (b) to examine differences in force-time metrics between starters and non-starters. Forty-two professional female handball players (e.g., SuperLeague) volunteered to participate in this study. Each athlete performed three maximum-effort CVJs with no arm swing while standing on a uni-axial force plate system sampling at 1,000 Hz. Independent *t*-tests were used to examine differences in each variable between starters and non-starters. The results revealed that starters attained superior performance within the eccentric phase of the CVJ when compared to non-starters, particularly in terms of eccentric peak velocity (−0.957 ± 0.242 vs. −0.794 ± 0.177 m·s^−1^), eccentric mean power (320.0 ± 77.7 vs. 267.1 ± 75.2 W), and eccentric peak power (929.0 ± 388.1 vs. 684.4 ± 214.2 W). While not reaching the level of statistical significance, moderate-to-large effect sizes were observed for concentric impulse, peak velocity, and mean and peak force and power, all in favor of players included in the starting lineup (*g *= 0.439–0.655). Overall, these findings suggest that at the top-tier level of handball competition, the ability to secure a spot in a starting lineup may be possibly influenced by the athlete's eccentric performance capabilities. Thus, the development of lower-body eccentric strength and power may positively impact on-court athlete performance and ultimately help the team secure the desired game outcome.

## 1 Introduction

Handball is a body-contact team sport with more than 19 million participants worldwide (1). As an official Olympic sport, the game involves two teams, each comprising six players and a goalkeeper (2). Defined by its fast-paced defensive and offensive actions, the main objective of the game is to score goals through strategic positioning, one-on-one actions, and ball passing (3). This requires players to constantly engage in high-intensity activities across short distances, including jumping, sprinting, and rapid change-of-direction movements (4, 5). In addition, players need to be proficient in performing handball-specific maneuvers such as throwing, passing, blocking, and checking in order to help the team secure the winning game outcome (3).

Considering the complex on-court competitive demands, handball players are required to possess a distinct set of physical and physiological attributes to attain peak performance levels (3). In terms of anthropometric characteristics, these values often differ across playing positions (6, 7). Several previously published research reports have found that wing players are shorter in stature and have lower body mass when compared to other players (8–10). Also, wing players tend to have superior endurance (11), jumping, and sprinting abilities when compared to backcourt players and pivots (12). However, it is important to note that regardless of position-specific differences, physical abilities such as endurance (4, 5), strength (13), power (6), running speed (14), and throwing velocity (15) are key factors that contribute to a successful handball performance.

The previously mentioned differences can further extend to comparing physical and physiological performance characteristics of athletes included in the starting lineup (i.e., starters) with their substitutions (i.e., non-starters) (16). Multiple research reports focused on examining athletes participating in team sports such as basketball (17, 18), volleyball (19, 20), and rugby (21), found superior neuromuscular performance attributes in starters than non-starters. Moreover, it should be noted that these differences have been particularly evident in female athletes, with starters demonstrating greater absolute and relative peak power production capacities (17, 22). However, in terms of handball-specific research, there is a notable gap in the scientific literature focused on examining neuromuscular performance characteristics, especially on the professional level of competition.

With rapid technological growth over the last decade, the countermovement vertical jump (CVJ) performed on portable force plate systems has become a gold-standard testing modality in an applied sports setting for the assessment of lower-body neuromuscular performance characteristics (23–26). It allows sports practitioners to obtain in-depth insight into multiple force-time metrics during both eccentric and concentric phases of the jumping motion with high levels of validity and reliability (25–27). Yet, despite jumping performance being one of the key physical performance attributes that handball players need to possess to proficiently perform sport-specific movements such as throwing and blocking (3), previous studies found no differences in jump height when examining elite and amateur male and female handball players (13, 28, 29). However, it is important to note that the aforementioned studies were solely focused on observing vertical jump height as an outcome metric, suggesting that CVJ performed on innovative force plate systems could provide a comprehensive understanding of lower-body neuromuscular performance capabilities.

Therefore, alongside a considerable lack of scientific literature pertaining to female athletes, the purpose of the present study was twofold: a) to provide a comprehensive CVJ profile of professional female handball players and b) to examine differences in force-time metrics during both eccentric and concentric phases of the CVJ between the players included in the starting lineup and their substitutions. Based on previously published research reports, it is hypothesized that starters are likely to demonstrate superior performance on multiple CVJ force-time metrics.

## 2 Materials and methods

### 2.1 Participants

Forty-two professional female handball players volunteered to participate in the present study (height = 173.5 ± 4.8 cm; body mass = 66.6 ± 7.1 kg; age = 22.0 ± 4.7 years), from which sixteen were starters (height = 174.5 ± 4.2 cm; body mass = 66.1 ± 4.2 kg; age = 24.0 ± 5.0 years) and twenty-four were non-starters (height = 172.9 ± 5.0 cm; body mass = 66.6 ± 8.6 kg; age = 20.6 ± 4.1 years). The cohort of participants encompassed athletes from three teams competing at the same level of play (e.g., SuperLeague). All athletes were active members of the team at the time point of the data collection and were cleared by their medical personnel to participate in team-specific activities. The testing procedures performed in this investigation were previously approved by the University's Institutional Review Board and all participants signed an informed consent document.

### 2.2 Procedures

Prior to the start of the CVJ testing procedures, all athletes completed a standardized warm-up protocol composed of dynamic stretching exercises (e.g., A-skips, butt-kicks, high knees, side-to-side lunges, high-knee-pulls) administered by their respective strength and conditioning coaches. Then, each athlete stepped on a uni-axial force plate system (ForceDecks Max, VALD Performance, Brisbane, Australia) sampling at 1,000 Hz and performed three CVJs with no arm swing (i.e., hands on the hips during the entire movement) (16, 26). Strong verbal encouragement to push the ground as forcefully as possible was provided through the testing procedures to ensure that each athlete was giving maximal effort (30). The system was re-calibrated between each participant, and the average values across three jumps were used for performance analysis purposes. To minimize the possible influence of fatigue, the rest between each jump trial was 10–15 s. All testing procedures were completed within the same time of the day (17:00–19:00 h) during the in-season competitive period, >48 h following the last game. In addition, following the completion of testing procedures, the athlete's age and height were obtained from the official team roster.

### 2.3 Variables

The selection of the dependent variables included in this investigation was based on previously published research reports that demonstrated adequate levels of validity and reliability (23–25, 31–33). The force-time metrics examined within the eccentric phase of the CVJ were: braking phase duration and impulse, eccentric duration, peak velocity, and mean and peak force and power. The force-time metrics examined during the concentric phase of the CVJ were: concentric duration, impulse, and peak and mean force and power. Also, jump height (i.e., impulse-momentum calculation), reactive strength index (RSI)-modified (i.e., jump height divided by contraction time), and countermovement depth were derived as CVJ outcome metrics. The reduction in system mass by 20 N was selected as the start of the contraction phase and it ended at take-off, which was determined by the reduction in vertical force below 20 N. The eccentric phase was defined as the phase with a negative center of mass velocity. In addition, the braking phase started at minimum force until the end of the eccentric phase, and the impulse was calculated as the area under the ground reaction force curve (24, 34).

### 2.4 Statistical analysis

Descriptive statistics, means and standard deviations (x̄ ± SD) were calculated for each dependent variable examined in the present study. Shapiro-Wilk test and Q-Q plots corroborated that the assumption of normality was not violated. Independent *t*-tests were used to examine statistically significant differences for each CVJ force-time metric of interest between starters (*n* = 16) and non-starters (*n* = 24). Due to the within-group sample sizes (*n* < 20), Hedges's *g* was calculated to provide the measure of the effect size (*g *= 0.2—small effect, *g *= 0.5—moderate effect, *g *= 0.8—large effect). Statistical significance was set *a priori* to *p *< 0.05. All statistical analyses were completed with SPSS (Version 26.0; IBM Corp., Armonk, NY, USA).

## 3 Results

Descriptive statistics for each CVJ force-time metric of interest and comparisons between starters and non-starters are presented in Table 1. Due to no statistically significant differences being found in body mass between the groups (*p *= 0.556; *g *= 0.187), the dependent variables were reported in absolute terms. The only statistically significant differences between starters and non-starters were found in eccentric peak velocity, eccentric peak power, and eccentric mean power. No other CVJ force-time metrics reached the level of statistical significance (*p *> 0.05).

**Table 1:** Countermovement vertical jump profile of professional female handball players and comparison between starters and non-starters.

Variable [unit]	All players	Non-starters	Starters	*p*-value	Effect size
Eccentric phase					
Braking phase duration [s]	0.294 ± 0.087	0.296 ± 0.089	0.290 ± 0.084	0.809	0.007
Eccentric braking impulse [N·s]	31.3 ± 10.1	29.4 ± 9.8	34.1 ± 10.2	0.153	0.472
Eccentric duration [s]	0.499 ± 0.102	0.507 ± 0.108	0.487 ± 0.096	0.540	0.193
Eccentric peak velocity [m·s^−1^]	−0.859 ± 0.218	−0.794 ± 0.177	−0.957 ± 0.242*	0.018	0.803
Eccentric peak force [N]	1,521.5 ± 240.3	1,461.9 ± 231.7	1,610.8 ± 231.7	0.054	0.643
Eccentric mean force [N]	660.4 ± 67.7	655.2 ± 78.7	668.2 ± 48.1	0.558	0.188
Eccentric peak power [W]	782.3 ± 315.8	684.4 ± 214.2	929.0 ± 388.1*	0.014	0.845
Eccentric mean power [W]	288.3 ± 79.6	267.1 ± 75.2	320.0 ± 77.7*	0.038	0.695
Concentric phase					
Concentric duration [s]	0.235 ± 0.039	0.237 ± 0.043	0.231 ± 0.035	0.660	0.028
Concentric impulse [N·s]	153.3 ± 18.6	149.6 ± 19.2	158.7 ± 16.7	0.130	0.496
Concentric peak velocity [m·s^−1^]	2.38 ± 0.16	2.35 ± 0.14	2.44 ± 0.18	0.103	0.578
Concentric peak force [N]	1,643.1 ± 211.1	1,602.3 ± 209.6	1,704.0 ± 204.4	0.136	0.489
Concentric mean force [N]	1,328.8 ± 141.3	1,304.2 ± 146.9	1,365.8 ± 127.8	0.180	0.439
Concentric peak power [W]	2,938.9 ± 374.1	2,871.8 ± 360.9	3,039.5 ± 382.4	0.168	0.455
Concentric mean power [W]	1,684.9 ± 255.8	1,621.0 ± 213.5	1,780.7 ± 289.8	0.052	0.655
Other					
Contraction time [s]	0.734 ± 131.5	0.744 ± 0.141	0.718 ± 0.117	0.538	0.196
Jump height [cm]	26.7 ± 4.0	25.8 ± 3.4	27.9 ± 4.6	0.107	0.542
RSI-modified [ratio]	0.376 ± 0.091	0.356 ± 0.089	0.407 ± 0.087	0.083	0.058
Countermovement depth [cm]	−21.6 ± 5.5	−20.8 ± 5.8	−22.8 ± 5.0	0.271	0.362

RSI, reactive strength index.

* significantly different when compared to non-starters (*p* < 0.05).

## 4 Discussion

Upon reviewing the existing scientific literature, we can confidently affirm that this is the first study focused on examining differences in neuromuscular performance characteristics between starters and non-starters within top-tier professional female handball players. It was hypothesized that athletes included in the starting lineup are likely to display superior performance on multiple CVJ force-time metrics when compared to their substitutions. The findings of the present investigation partially align with the aforementioned hypothesis as starters did demonstrate a significantly greater eccentric peak velocity and eccentric mean and peak power. However, no significant differences were observed within the concentric phase of the CVJ as well as in outcome or strategy metrics such as vertical jump height and countermovement depth.

The obtained results are similar to some of the previously published studies comparing starters and non-starters in other team-based sports such as basketball (17, 18), volleyball (19, 20), soccer (22, 35), rugby (21) and lacrosse (36). Overall, the results of the aforementioned research reports implied superior neuromuscular performance characteristics of players included in a starting lineup when compared to their substitutions. In the context of female athletes, Becerra-Patino et al. (37) and Riley et al. (38) found that starters in soccer tend to have higher vertical jump heights than their substitutions (38.6 ± 6.1 vs. 34.4 ± 3.8 cm, respectively). Additionally, when examining a cohort of NCAA Division-I female basketball players, Gonzales et al. (17) found that starters are likely to experience improvements in both vertical jump and squat performance throughout the competitive season span, while no difference was observed within their non-starter counterparts. Specifically, starters attained greater absolute and relative peak power during the vertical jump test as well as higher average squat power (17). In addition, it is worth noting that the lack of significant differences in vertical jump height observed in the present investigation aligns with previous findings involving elite female handball players (28, 29). Thus, it can be inferred that jump height as a sole performance indicator might not be able to provide practitioners with useful information that can be used to distinguish between players based on their performance capabilities, further emphasizing the importance of an in-depth analysis approach that includes both eccentric and concentric phases of the CVJ.

When interpreting phase-specific differences in CVJ performance, it can be observed that starters had greater eccentric mean and peak power, accompanied by a greater eccentric peak velocity. Large effect sizes were observed for eccentric peak power and velocity (*g *= 0.803–0.845), while eccentric mean power demonstrated a moderate-to-large effect size magnitude (*g *= 0.695). Considering that *power = force × velocity (*39), these results imply that superior eccentric power observed within the athletes included in the starting lineup is primarily attained by an increase in eccentric velocity rather than eccentric force. Also, while eccentric force remains an important factor, in this instance successful performance may be more contingent on the ability to exert a certain amount of force at a maximal velocity, which resembles the on-court competitive demands (40). Furthermore, Spiteri et al. (41) highlighted a considerable impact of eccentric strength on the overall athlete's strength profile. A strong association (r = −0.79–0.89) was found between multiple eccentric force-time metrics and performance on change-of-direction tests (i.e., *T*-test and 505 test) within a cohort of NCAA Division-I basketball players (42). These results further confirm the findings from a recently published study involving professional female handball players, which revealed a positive relationship between CVJ neuromuscular performance metrics and horizontal deceleration performance, encompassing maximal and average deceleration and maximal approach velocity (2). Combined, these results underscore the importance of the eccentric phase of CVJ in the execution of various handball-specific movements. Therefore, given the intermittent multidirectional nature of the handball game (3), it can be assumed that players with superior eccentric muscle qualities can perform offensive and defensive actions more effectively, particularly those that require players to decelerate quickly to create elevation for jump throws or change movement direction to create space for strategic ball positioning. Therefore, such players are more likely to secure a spot in a team's starting line-up.

Despite not reaching the level of statistical significance, it is still important to recognize that the differences in the majority of force-time metrics between starters and non-starters within the concentric phase of the CVJ were moderate in magnitude (*g *= 0.439–0.655). Specifically, concentric impulse, peak velocity, and mean and peak force and power were slightly greater for starters when compared to non-starters. When taking into account that both groups had almost identical body mass, we can assume that a development of CVJ concentric strength and power capabilities cannot be underestimated and may still offer a competitive edge in certain instances that may help player secure the spot in starting lineup (e.g., establishing a better position on the court during body-contact offensive actions). Also, it should be noted that the magnitudes of the aforementioned force-time metrics align with the ones recorded in similar studies conducted on elite female handball players (2), suggesting that attaining the observed levels of strength and power is a required to compete on this level of play. Thus, analyzing the concentric phase of CVJ can still be beneficial when determining the starting team lineup, especially considering that maximal strength provides the foundation for developing all other components of strength, including eccentric strength and power (42).

Despite offering valuable insight into the neuromuscular performance characteristics of professional female handball players, this study is not without limitations. The cohort of participants was solely based on studying female athletes, which may limit the applicability of the observed findings to male athlete counterparts. Also, future research is warranted to examine if these findings apply to other competitive levels (e.g., amateur or Olympic). In addition, further research is warranted to examine the impact of maturation status, tactical-technical knowledge of the game, and playing position on differentiating between players included in the starting lineup and their substitutions.

In summary, the findings of the present study reveal that the ability to secure a spot in the starting lineup at the professional level of female handball competition may be influenced by an athlete's eccentric performance characteristics (i.e., eccentric peak velocity and eccentric mean and peak power). Also, alongside providing normative ranges for CVJ performance assessment that coaches, sports scientists, and strength and conditioning practitioners can use when developing training regimens, these results further emphasize the importance of the development of lower-body eccentric strength and power characteristics. When considering the on-court competitive demands, they may positively impact various aspects of the game such as throwing velocity, blocks, throws, and holds, and ultimately improve overall team performance and increase the likelihood of securing the desired game outcome.

## Data Availability

The raw data supporting the conclusions of this article will be made available by the authors, without undue reservation.
